# Comparing Glucose Outcomes Following Face-to-Face and Remote Initiation of Flash Glucose Monitoring in People Living With Diabetes

**DOI:** 10.1177/19322968231176531

**Published:** 2023-05-25

**Authors:** Andrew P. Kingsnorth, Caroline Wilson, Pratik Choudhary, Tomás P. Griffin

**Affiliations:** 1 Diabetes Research Centre, University of Leicester, Leicester General Hospital, Leicester, UK; 2 NIHR Leicester Biomedical Research Centre, Leicester, UK; 3 School of Medicine, University of Limerick, Limerick, Ireland

**Keywords:** continuous glucose monitoring, onboarding, FreeStyle Libre, type 1 diabetes, remote, virtual

## Abstract

**Background:**

When launched, FreeStyle Libre (FSL; a flash glucose monitor) onboarding was mainly conducted face-to-face. The COVID-19 pandemic accelerated a change to online starts with patients directed to online videos such as Diabetes Technology Network UK for education. We conducted an audit to evaluate glycemic outcomes in people who were onboarded face-to-face versus those who were onboarded remotely and to determine the impact of ethnicity and deprivation on those outcomes.

**Methods:**

People living with diabetes who started using FSL between January 2019 and April 2022, had their mode of onboarding recorded and had at least 90 days of data in LibreView with >70% data completion were included in the audit. Glucose metrics (percent time in ranges) and engagement statistics (previous 90-day averages) were obtained from LibreView. Differences between glucose variables and onboarding methods were compared using linear models, adjusting for ethnicity, deprivation, sex, age, percent active (where appropriate), and duration of FSL use.

**Results:**

In total, 935 participants (face-to-face 44% [n = 413]; online 56% [n = 522]) were included. There were no significant differences in glycemic or engagement indices between onboarding methods and ethnicities, but the most deprived quintile had significantly lower percent active time (b = −9.20, *P* = .002) than the least deprived quintile.

**Conclusions:**

Online videos as an onboarding method can be used without significant differences in glucose and engagement metrics. The most deprived group within the audit population had lower engagement metrics, but this did not translate into differences in glucose metrics.

## Introduction

The FreeStyle Libre (FSL; Abbott, Abbott Park, IL) is a type of continuous glucose monitoring (CGM) device known as a flash glucose monitor because data are only transferred from the sensor when it is scanned by a reader (the FSL 1/2 reader or FreeStyle LibreLink app on a smartphone). This obviates the need for fingerpricks which removes the pain, inconvenience, and anxiety associated with self-monitoring of blood glucose measurements.^1-3^ The original FSL1 did not have alarms, whereas the newer FSL2 has optional alarms that are customizable for low and high blood glucose levels. There are robust real-world and trial data supporting the use of flash glucose monitoring as it has been shown to improve glycated hemoglobin (HbA1c), time in range (TIR), hypoglycemic unawareness and decrease diabetes distress and hospitalization from acute diabetes complications.^4-9^ Similarly, the FLASH-UK trial showed that using flash glucose monitoring compared with fingerstick testing safely decreased HbA1c by 0.5% and increased TIR by 9%.^4^ In light of these data, CGM is now recognized in national and international guidelines as a key pillar in the self-management of type 1 diabetes (T1DM).^10,11^ Current National Institute for Health and Care Excellence guidelines recommend that all adults with T1DM should be offered a choice of CGM, based on that individual’s preferences and needs and the characteristics of the devices available.^11^ The use of CGMs in type 2 diabetes (T2DM) is evolving, but those with recurrent or severe hypoglycemia, impaired hypoglycemia awareness, or who would be advised to monitor blood glucose levels more than or equal to eight times a day should also be offered CGM.^12^

Due to the COVID-19 pandemic which resulted in mandatory lockdowns and social distancing, there was an enforced, accelerated change in how diabetes care was delivered both locally, nationally, and internationally with care transitioning from predominantly face-to-face to online. The urgent clinical need during the pandemic to support more people living with diabetes and to help optimize glycemic control coincided with expanding access criteria for flash glucose monitoring. This combined with the shift to virtual care mandated that health care professionals (HCPs) explored and developed the relatively new concept of predominantly self-directed online education for diabetes technologies. However, it is unclear from the current available literature if there is a difference in glycemic outcomes between face-to-face and self-directed online flash glucose monitoring onboarding. Therefore, the aim of this audit was to determine the impact of different methods of onboarding on glycemic outcomes and flash glucose monitoring engagement metrics in people living with diabetes, while controlling for key demographic factors that may impact these outcomes.

## Methods

A clinical audit was conducted to evaluate the impact of the change from face-to-face to self-directed online onboarding of flash glucose monitoring within the secondary care setting of Leicester General Hospital. The audit was registered and approved by the Clinical Audit Facilitator Team at University Hospitals of Leicester NHS Trust. All patients that had been prescribed the FSL before April 2022 with a known method of onboarding (either face-to-face or online) were eligible for inclusion. Data used in this audit were collected as part of routine clinical practice, and once extracted, the data were anonymized before analyses, and as such, no ethical approval was required.

### Onboarding Procedure

Training for use of CGM and continuous subcutaneous insulin infusion in the past was provided 1:1 or in small groups. Tyndall et al demonstrated that large face-to-face group onboarding sessions were effective with a reduction in HbA1c of 0.6%.^13,14^ In Leicestershire, face-to-face onboarding groups comprised of 15 to 25 patients with two hours of education delivered by trained HCPs. Training involved discussions on the use of trend arrows, LibreView (Abbott), and carbohydrate counting. For those for whom a group start was deemed inappropriate or in whom a group start was unsuccessful, individual patient education was provided.

In contrast, patients who were onboarded online were invited to self-review the Libre Academy (https://progress.freestylediabetes.ie/) or Diabetes Technology Network UK (DTN-UK; https://abcd.care/dtn-education/flash-glucose-monitoring) modules online. Once the relevant documentation was completed, a link was provided to order the sensor and reader (if not, using the FSL phone application). Although it was encouraged for patients to view the modules, proof of completion was not required.

### Measurements

#### Glucose metrics and usage statistics

As a part of routine clinical care, data from the FSL are shared by the person living with diabetes via cloud infrastructure called LibreView (Abbott). At the time this audit was undertaken, the original FSL1 was being phased out at our center, and >80% of participants at our center were on FSL2. We accessed LibreView 90 days after the last person included in the audit was onboarded and gathered the most recent glucose metrics indices and usage statistics (90-day averages) for each participant: average glucose, percent TIRs (below range [<3.9 mmol/L], in range [3.9-10.0 mmol/L], above range [>10.0 mmol/L]), and usage statistics (average sensor active time and number of scans per day). To be included within the analyses of glucose metrics, participants needed to have ≥70% usage data over the 90-day monitoring period, but all participants were included in analyses of FSL usage statistics.

#### Demographics and clinical indices

We extracted demographics, diabetes status, and postcode from electronic health records (SystmOne; TPP, Horsforth, UK), and Index of Multiple Deprivation (IMD) deciles were collected using the IMD Postcode Checker.^15^ To reduce the number of comparison groups, ethnicity codings from SystmOne (TPP) were recoded into Asian, Black, Mixed, Other, and White, and IMD deciles were reduced to five groupings. We obtained information regarding the flash glucose monitoring onboarding method (face-to-face or online) and onboarding date from a prospectively maintained CGM database. Age was also divided by 10 to create more meaningful coefficients within the models. To conduct a sensitivity analysis into whether there were any differences in the change of HbA1c values between onboarding groups, measurements corresponding to the start date and audit date for patients were also extracted via SystmOne (TPP) and iLab (iSOFT, Sydney, Australia). Patients who had been on Libre for at least three months were selected, with matched HbA1c values from up to six months before and up to six months after the audit date. All data were merged by NHS number, anonymized by the study team, and then analyzed within R version 4.1.3 (R Foundation for Statistical Computing, Austria).

### Statistical Analyses

Unadjusted values for demographic and outcome variables were assessed for statistical differences between groups. Multiple linear regression models were also used to explore differences between TIRs and onboarding method. Models were adjusted for ethnicity, IMD groupings, age, sex, the length of FSL duration (number of days between onboarding and audit date), and the percent of active data. All analyses were conducted using R, and model statistical significance was set at *P* < .01 to account for multiple comparisons.

## Results

### Clinical Characteristics

Complete FSL, onboarding, and demographic data were available for 1261 individuals. This decreased to 935 (74.2%) individuals after removing those with <70% active time (throughout the 90-day period). The characteristics of the study participants (≥70% data) are described in Table 1, and the entire unfiltered sample split by onboarding method and by data completeness is included within the Supplementary Material (Tables S1 and S2). Of those included, 44% and 56% were onboarded face-to-face and online, respectively. Patients were a median (interquartile range) of 47 (34-58) years old and predominantly male (52%), had T1DM (83%), and were of white ethnicity (59%). Although 12% of participants were recorded as having not specified diabetes or other type of diabetes, we expect that the majority of these participants have T1DM that has not been coded correctly because NHS-funded access for flash glucose monitoring at the time of the audit was predominantly for people living with T1DM. Between onboarding methods, online patients were significantly younger (46 [34, 56] vs 50 [35, 60], *P =* .016), and there were a greater proportion of individuals coded as having T2DM and within more deprived quintile groupings.

**Table 1.** Descriptive Characteristics of the Study Sample.

Characteristics	All (n = 935)	Face-to-face (n = 413)	Online (n = 522)	*P*
Age in years, median (IQR)	47.0 (34.0, 58.0)	50.0 (35.0, 60.0)	46.0 (34.0, 56.0)	.**016**
Sex, n (%)				.15
Female	446 (47.7%)	208 (50.4%)	238 (45.6%)	
Male	489 (52.3%)	205 (49.6%)	284 (54.4%)	
Diabetes type, n (%)				.**005**
T1DM	772 (82.6%)	357 (86.4%)	415 (79.5%)	
T2DM	49 (5.2%)	12 (2.9%)	37 (7.1%)	
Not specified and other	114 (12.2%)	44 (10.7%)	70 (13.4%)	
Ethnicity, n (%)				.5
Asian	114 (12.2%)	45 (10.9%)	69 (13.2%)	
Black	13 (1.4%)	4 (1.0%)	9 (1.7%)	
Mixed	237 (25.3%)	113 (27.4%)	124 (23.8%)	
Other	24 (2.6%)	11 (2.7%)	13 (2.5%)	
White	547 (58.5%)	240 (58.1%)	307 (58.8%)	
Deprivation score, n (%)				.**020**
Deprivation group 1 (most)	71 (7.6%)	22 (5.3%)	49 (9.4%)	
Deprivation group 2	133 (14.2%)	50 (12.1%)	83 (15.9%)	
Deprivation group 3	158 (16.9%)	65 (15.7%)	93 (17.8%)	
Deprivation group 4	285 (30.5%)	137 (33.2%)	148 (28.4%)	
Deprivation group 5 (least)	288 (30.8%)	139 (33.7%)	149 (28.5%)	
Libre metrics				
Libre duration in days, median (IQR)	656.0 (394.0, 946.0)	974.0 (891.0, 1,101.0)	424.0 (281.2, 575.7)	**<.001**
Percent active time, median (IQR)	95.0 (89.0, 99.0)	96.0 (90.0, 100.0)	95.0 (88.0, 99.0)	.**032**
Scans per day, n (%)	10.0 (7.0, 14.0)	11.0 (8.0, 15.0)	10.0 (7.0, 14.0)	.**038**
Average glucose (mmol/L), median (IQR)	9.8 (8.6, 11.1)	9.6 (8.6, 11.1)	9.8 (8.6, 11.2)	.3
Glucose management indicator (%), median (IQR)	7.5 (7.0, 8.1)	7.4 (7.0, 8.1)	7.5 (7.0, 8.1)	.3
Coefficient of variation (%), median (IQR)	37.9 (34.3, 42.2)	37.9 (34.7, 42.0)	38.0 (34.0, 42.5)	.7
Percent time in ranges				
Below range (<3.9 mmol/L), median (IQR)	2.0 (1.0, 5.0)	2.0 (1.0, 5.0)	2.0 (1.0, 4.8)	.**043**
In range (3.9-10.0 mmol/L), mean (SD)	53.5 (18.0)	54.0 (17.2)	53.1 (18.7)	.4
Above range (>10.0 mmol/L), median (IQR)	43.0 (30.0, 56.0)	42.0 (30.0, 56.0)	44.0 (29.2, 57.0)	.3
Glycated hemoglobin (HbA1c)^a^				
Start HbA1c (mmol/mol), median (IQR)	66.0 (56.0, 76.0)	66.0 (57.0, 73.0)	66.0 (54.0, 79.0)	.6
Audit HbA1c (mmol/mol), median (IQR)	59.0 (52.0, 68.0)	58.0 (53.0, 68.0)	60.0 (51.0, 68.0)	.9
Change in HbA1c (mmol/mol), median (IQR)	−4.5 (−13.0, 2.0)	−4.0 (−12.0, 2.0)	−6.0 (−14.0, 2.0)	.3

Not specified was the coding entry “Diabetes—not specified” within SystmOne, and “Other” equals any other subtype of diabetes. *P* values indicate statisitical differences between onboarding methods, and bold *P* values indicate *P* < .05.

Abbreviations: IQR, interquartile range; SD, standard deviation; T1DM, type 1 diabetes mellitus; T2DM, type 2 diabetes mellitus.

a Matched data available for 324 patients (163 face-to-face and 161 online)

**Figure 1.**
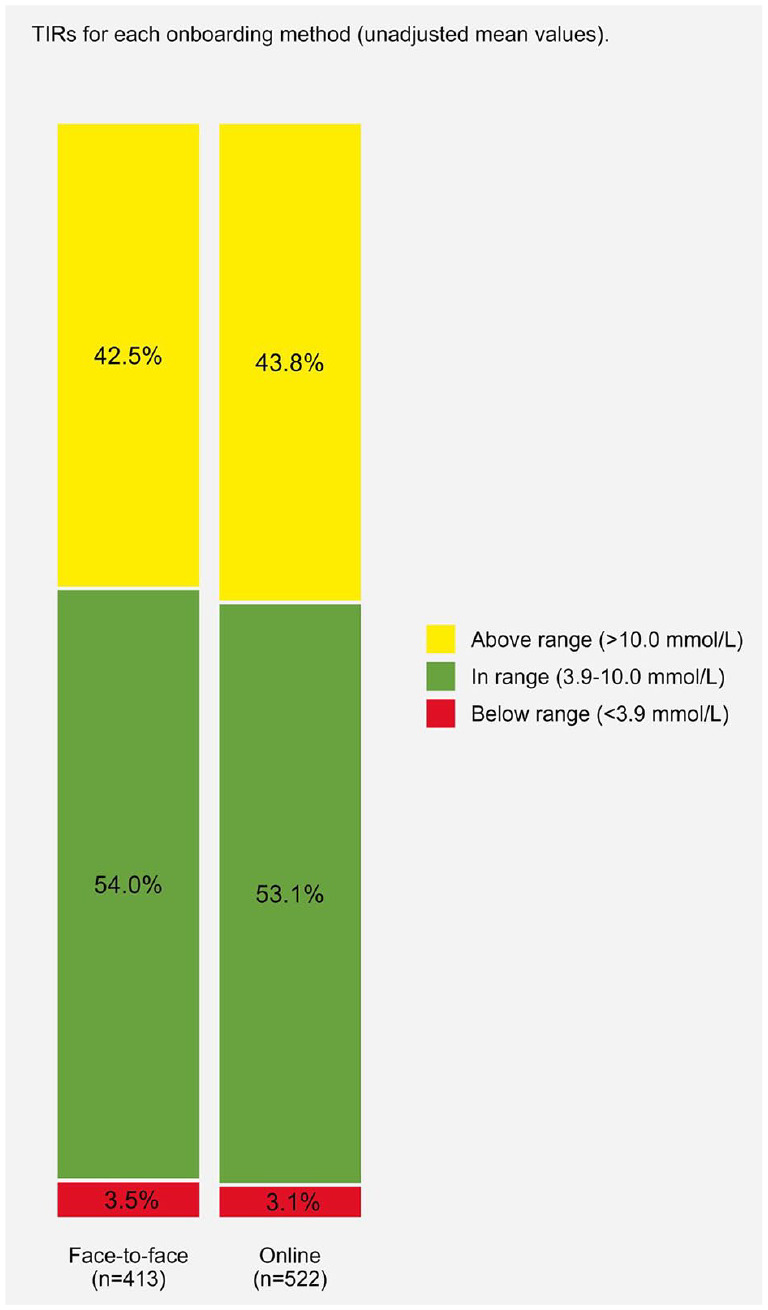
Unadjusted time in ranges (TIRs) for each onboarding group.

Patients had, on average, 53.5% (±18%) time within range (3.9-10.0 mmol/L), which is comparable (54.5%) to that of a similar-aged UK-based cohort assessed during a similar period of COVID,^9^ and had a median of 95.0% (89.0%, 99.0%) active time. Those who were onboarded online had fewer days of Libre usage (424.0 [281.2, 575.7] vs 974.0 [891.0, 1101.0] days, *P* < .001), lower percent active time (95.0 [88.0, 99.0] vs 96.0 [90.0, 100.0], *P* = .032), and fewer scans per day (10.0 [7.0, 14.0] vs 11.0 [8.0, 15.0], *P* = .038). Time below range was statistically but not clinically different, and no other glucose metrics were statistically significant between onboarding methods. When all participants were included (both <70% and ≥70% usage), the sample had a median of 91.0% (68.0, 98.0) active time and 8.0 (5.0, 12.0) scans per day. Unadjusted mean time in glucose ranges for both onboarding methods is displayed within Figure 1 and, including level 2 ranges, within Supplementary Material Figure S1.

**Figure 2.**
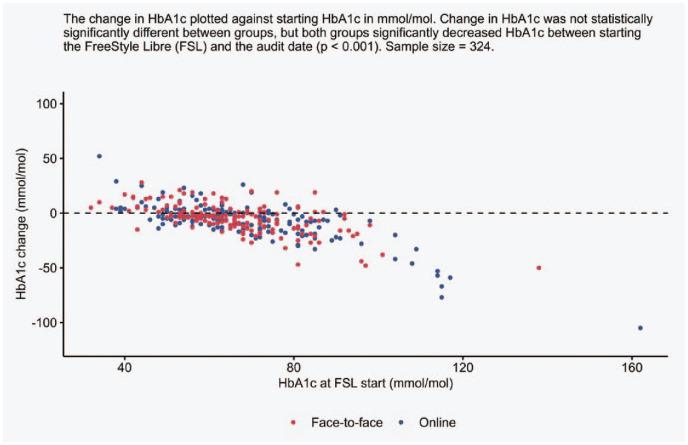
Changes in glycated hemoglobin (HbA1c) between FreeStyle Libre (FSL) start and the audit date for both onboarding methods.

Matched HbA1c values were available for 324 patients, which reflects that routine measurements were paused during the pandemic. There was a statistically significant reduction of −4.5 mmol/mol from baseline to the last measured HbA1c in both groups (*P* < .001), with no differences in start, end, or change between groups. Changes in HbA1c per starting HbA1c value for both groups are represented within Figure 2.

### Adjusted Analyses

**Figure 3.**
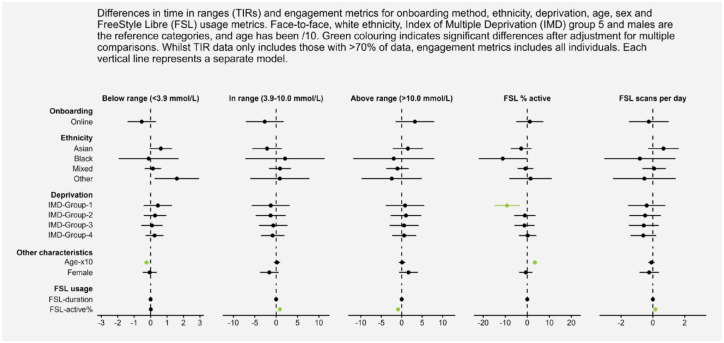
A forest plot representing the coefficients for onboarding, ethnicity, deprivation, age, sex, and FSL usage for all time in range and engagement metrics. Abbreviations: IMD, Index of Multiple Deprivation; FSL, FreeStyle Libre.

The results of multiple linear regressions assessing differences between onboarding methods while accounting for demographic variables are displayed within the forest plot of Figure 3. The onboarding method was not a significant predictor, indicating that glycemic and engagement metrics did not differ by onboarding method within this sample. Ethnicity was not significantly associated with glycemic and engagement metrics. However, those in the most deprived group had significantly lower percent active time (b = −9.20, *P* = .002) than those in the least deprived group if all other variables were held constant. Increasing age was significantly associated with lower percent time below the range (b = −0.25, *P* < .001, coefficient is age/10) but greater percent active time (b = 3.47, *P* < .001). Duration of FSL use was not significantly associated with glycemic or engagement metrics. However, greater percent active time was associated with more TIR (0.86, *P* < .001) and less time spent above range (b = −0.88, *P* < .001), while controlling for all other variables.

## Discussion

To our knowledge, this is the first published comparison of face-to-face versus online onboarding of flash glucose monitoring in a large tertiary referral center. Overall, the method of onboarding had no statistically significant impact on glucose metrics or usage statistics. However, those in the most deprived group have significantly lower FSL percent active time than those in the least deprived group. This did not translate into differences in TIR in our analyses; which we suggest is related to our data-selection criteria (≥70% of data). Age and percent active time of the FSL were shown to be related to TIR and engagement metrics, but the differences are unlikely to be clinically relevant.

In the Association of British Clinical Diabetologists (ABCD) Nationwide Audit exploring the effect of flash glucose monitoring (FSL1—no optional alarms) on glycemic control, hypoglycemia, diabetes-related distress, and resource utilization, 89% of participants with follow-up data used flash glucose monitoring >70% of the time.^5^ In our audit, only 74.6% of participants used flash glucose monitoring ≥70% of the time, with no difference between methods of onboarding. All participants with data regarding onboarding attending our center were included, but in the ABCD Nationwide Audit, data collection was biased toward individuals who had follow-up visits as a part of routine clinical care, so a direct comparison is not possible. For those with both follow-up HbA1c and TIR in the ABCD Nationwide Audit, the median TIR was 43% (27%-56%), which is lower than the 53.3% (18.0%) in our study. In addition, the FSL1 was the flash glucose monitor used in the ABCD Nationwide Audit, whereas FSL2 (customizable high and low alarms) was used in >80% of participants in our study. Our results are comparable to those reported by Leelarathna et al in their parallel-group multicenter randomized controlled trial involving FSL2 (%TIR: 52.1% ± 15.9%) in people with T1DM.^4^ Comparable results were seen in Belgium^16^ and in a real-world evidence study that evaluated the impact of lockdown on glycemic measures.^9^ In a Polish study, however, higher TIR (64.2%) was observed, but participants were scanning on average 21 times a day.^8^

Although only 83% of our cohort are documented as having T1DM, we expect that the vast majority of those classed as type of diabetes not specified or other also have T1DM. This would be in line with the funding criteria that were in place in the NHS for flash glucose monitoring at the time of the audit,^11^ with ongoing broadening of criteria for access to flash glucose monitoring for people with T2DM.^12^ In terms of usage, patients on average scanned their FSL 10 times per day (whole cohort, eight scans without data restrictions), with no difference observed between onboarding methods. This number is substantially lower than the 16.3 scans per day reported across seven European countries including the United Kingdom, which is important as people who scan more frequently have increased TIR and decreased time in hyperglycaemia.^17^ This is supported somewhat in our audit as we found that patients who had greater percent active time, and therefore would signify greater scanning, had greater TIR and fewer percent above range.

There are over 400 000 people with T1DM (increasing at a rate of 4% per year) and more than 4.9 million people with a diagnosis of diabetes in the United Kingdom.^18,19^ Adoption of new technologies on such a large scale will be challenging; however, our study has shown that the resource-intensive nature of in-person training may not be required for device starts. This is important as face-to-face requires ongoing access to physical infrastructure and may delay the opportunity for the person to access the technology by being placed on a wait-list. Online onboarding could reduce the decision to deployment time. Importantly, the time and space maybe better used by the HCP to deliver education and support to those who need it most. Ease of access and combined with granular flash glucose monitoring and new methods of recording insulin delivery such as smart connected devices will facilitate the delivery of data-driven consultations that will both improve the quality of a consultation and optimize outcomes for the person living with diabetes.

Tyndall et al have shown the benefit of large-group flash glucose monitoring starts with one-hour education delivered as a lecture and follow-up as a part of routine clinical care on glycemic outcomes.^13^ This suggests that some of the value from flash glucose monitoring maybe having access to the device itself and, thus, easy access to glucose data at a glance rather than from the education surrounding the onboarding process. Our audit is reassuring in that it has shown no significant differences in glycemic outcomes between online and face-to-face onboarding, with little to no differences in glycemic outcomes between those of different ethnicities and socioeconomic status. This builds confidence that flash glucose monitoring rollout via remote onboarding in primary rather than tertiary care is feasible and effective. In particular, it offers people living with diabetes a more convenient and efficient way to access this transformative technology in a manner that can be readily adopted into their daily lives with minimal inconvenience. Although patients were provided with links to Libre Academy and/or DTN-UK modules, we do now know how many people availed these online resources, and from clinical experience, we know that many people did not.

Although our audit did not find any difference in TIR between ethnicity and deprivation categories, it is worth noting that 8% of participants are in the lowest most deprived quintile compared with 31% of participants in the upper least deprived quintile. Lower socioeconomic status, independent of HbA1c, is a risk factor for the development of diabetic retinopathy in people with T1DM^20^ and foot ulceration in people with T1DM and T2DM.^21^ Based on the NHS Diabetes Audit, the use of CGM is more prevalent among adults in the least deprived quintiles than among those in the most deprived quintiles.^22^ Also, among 1631 adults living with T1DM in three urban hospitals in the United Kingdom, the highest use of diabetes technology (continuous subcutaneous insulin infusion, real-time CGM, or flash glucose monitoring) was among the least deprived quintile (67% of those in the least deprived quintile vs 45% in the most deprived quintile).^23^ Within our audit, the proportion of participants accessing online onboarding increased in the lower deprivation groups than those accessing face-to-face onboarding (IMD group 1: 9.4% versus 5.3%; IMD group 2: 15.9% versus 12.1%). Importantly, initiation of technology has a positive impact on glycemic control irrespective of the deprivation quintile.^23^ We found that in those with FSL usage ≥70%, there were no differences in glycemic outcomes between the quintiles. We hypothesize that those in greater deprivation groups may not always be in a position to attend physical onboarding because of issues with transportation or the inability to take time off from work (physical onboarding is typically done during the routine HCP working day). Therefore, online onboarding can allow the person to do it in their own environment and, as a key factor, in their own time. However, in the total cohort, FSL usage was lower in the most deprived cohort than in the least deprived cohort. This suggests that a novel educational/training package (automated reminders, bespoke training, or virtual drop-in sessions) maybe beneficial to highlight the potential impact of increased flash glucose monitoring usage on glucose metrics and outcomes.

Although the method of onboarding did not appear to cause statistically significant differences in outcomes when controlling for key demographics, this does not mean that online onboarding will suit all people. Therefore, although our data support a choice, it remains imperative that centers retain the capacity of both onboarding modalities. In a study of 16 English-speaking parents of 15 children who initiated glucose monitoring technologies via telehealth within 30 days of diagnosis during the COVID-19 pandemic, parents found online onboarding very convenient, easy to do, and integrated into day-to-day life while at home.^24^ However, a smaller number of parents found the online environment intimidating, challenging, and overwhelming, and study participants concluded that it is important for clinics to offer a choice of face-to-face and online CGM onboarding.^24^ Important considerations when determining if a person is suitable for an online onboarding should include digital literacy, device-specific requirements, comorbidities, and of course, an individual’s preference.^25^ Approximately 11.9 million people in the United Kingdom do not have the necessary digital skills needed for everyday life.^26^ Consequently, not offering a face-to-face onboarding option has the potential to foster inequality in access to novel diabetes technologies between those with and those without digital literacy skills.

### Strengths and Limitations

The primary aim of this research study was to determine if onboarding method impacted glucose and engagement metrics, which was made possible by the usage of routinely collected flash glucose monitoring data. To our knowledge, this is one of the first analyses to demonstrate that there were no differences in glycemic outcomes or usage statistics between those who onboard flash glucose monitoring face-to-face and those via online means, which is a strength. However, there are several limitations to this audit that should be highlighted. There was a significant difference in the length of time between onboarding methods, which reflects the changing landscape due to COVID-19. Although we have controlled for FSL usage within our models, onboardings since mid-2020 have predominantly been conducted online. It is therefore unknown whether the online group represents lower risk patients and not those with potentially poorer outcomes due to delayed presentation related to COVID-19, and so our models may be influenced by implicit bias. Also, overtime patients and HCPs have become more skilled in the interpretation of FSL data. HCPs have fostered skills in supporting patients achieve optimal outcomes using FSL. Data regarding change in HbA1c were available but only in a small proportion of patients as during the pandemic, only essential blood tests were requested, and we grew accustomed to relying on glucose management indicator and TIR for clinical care. Type of diabetes was not recorded for 12% of participants, and the proportion of participants with T2DM is low and would warrant analysis in a larger cohort to make generalizable conclusions. FSL sensor version was also not captured, but patients were predominantly on the FSL2 sensor during the time of the audit.

## Conclusions

For people living with diabetes, there were no clinically significant differences in glycemic outcomes or engagement indices between those onboarded face-to-face and those via online means. Our results build confidence that flash glucose monitoring can be rolled out in primary care via online methods, to be done at a time and place convenient for the person. However, it is important that we continue to offer both face-to-face and online onboarding of flash glucose monitoring to ensure equal access to care, for those that would like additional support when starting their therapy.

## Supplemental Material

sj-docx-1-dst-10.1177_19322968231176531 – Supplemental material for Comparing Glucose Outcomes Following Face-to-Face and Remote Initiation of Flash Glucose Monitoring in People Living With Diabetes Click here for additional data file. Supplemental material, sj-docx-1-dst-10.1177_19322968231176531 for Comparing Glucose Outcomes Following Face-to-Face and Remote Initiation of Flash Glucose Monitoring in People Living With Diabetes by Andrew P. Kingsnorth, Caroline Wilson, Pratik Choudhary, Tomás P. Griffin and Dip Clin Ed in Journal of Diabetes Science and Technology
