# Supplementary material for: Comparing Glucose Outcomes Following Face-to-Face and Remote Initiation of Flash Glucose Monitoring in People Living With Diabetes
Source: J Diabetes Sci Technol. 2023 May 25;17(4):887–94. doi: 10.1177/19322968231176531 (PMC10347994; doi:10.1177/19322968231176531)
Supplement: sj-docx-1-dst-10.1177_19322968231176531 – Supplemental material for Comparing Glucose Outcomes Following Face-to-Face and Remote Initiation of Flash Glucose Monitoring in People Living With Diabetes [file sj-docx-1-dst-10.1177_19322968231176531.docx]

# Supplementary material

| **Table S1:** Descriptive characteristics of the entire sample split by onboarding method. | | | |  |
| --- | --- | --- | --- | --- |
|  | **All**  **(n = 1261)** | **Face-to-face**  **(n = 560)** | **Online**  **(n = 701)** | ***p*** |
| **Age in years, median (IQR)** | 45.0 (32.0, 57.0) | 46.0 (33.0, 59.0) | 44.0 (32.0, 55.0) | **0.02** |
| **Sex, n (%)** |  |  |  |  |
| Female | 615 (48.8%) | 285 (50.9%) | 330 (47.1%) | 0.2 |
| Male | 646 (51.2%) | 275 (49.1%) | 371 (52.9%) |  |
| **Diabetes type, n (%)** |  |  |  | 0.06 |
| T1DM | 1,043 (82.7%) | 475 (84.8%) | 568 (81.0%) |  |
| T2DM | 64 (5.1%) | 20 (3.6%) | 44 (6.3%) |  |
| Not specified & other | 154 (12.2%) | 65 (11.6%) | 89 (12.7%) |  |
| **Ethnicity, n (%)** |  |  |  | 0.2 |
| Asian | 161 (12.8%) | 64 (11.4%) | 97 (13.8%) |  |
| Black | 25 (2.0%) | 6 (1.1%) | 19 (2.7%) |  |
| Mixed | 316 (25.1%) | 145 (25.9%) | 171 (24.4%) |  |
| Other | 32 (2.5%) | 16 (2.9%) | 16 (2.3%) |  |
| White | 727 (57.7%) | 329 (58.8%) | 398 (56.8%) |  |
| **Deprivation score, n (%)** |  |  |  | **0.01** |
| Deprivation group 1 (most) | 118 (9.4%) | 41 (7.3%) | 77 (11.0%) |  |
| Deprivation group 2 | 185 (14.7%) | 70 (12.5%) | 115 (16.4%) |  |
| Deprivation group 3 | 226 (17.9%) | 95 (17.0%) | 131 (18.7%) |  |
| Deprivation group 4 | 364 (28.9%) | 172 (30.7%) | 192 (27.4%) |  |
| Deprivation group 5 (least) | 368 (29.2%) | 182 (32.5%) | 186 (26.5%) |  |
| **Libre metrics** |  |  |  |  |
| Libre duration in days, median (IQR) | 657.0 (394.0, 950.0) | 974.0 (891.0, 1,101.0) | 420.0 (282.0, 583.0) | **<0.001** |
| Percent active, median (IQR) | 91.0 (68.0, 98.0) | 92.0 (68.0, 99.0) | 90.0 (68.0, 98.0) | 0.3 |
| Scans per day, n (%) | 8.0 (5.0, 12.0) | 9.0 (5.0, 13.0) | 8.0 (4.0, 12.0) | 0.2 |
| Average glucose (mmol/L), median (IQR) | 10.0 (8.7, 11.7) | 9.9 (8.8, 11.6) | 10.1 (8.7, 11.8) | 0.4 |
| Glucose Management Indicator (%), median (IQR)^a^ | 7.6 (7.1, 8.4) | 7.6 (7.1, 8.3) | 7.6 (7.0, 8.4) | 0.4 |
| Coefficient of variation (%), median (IQR) | 38.5 (34.5, 43.0) | 38.8 (35.1, 42.6) | 38.3 (34.0, 43.3) | 0.5 |
| Percent time in ranges |  |  |  |  |
| Below range (<3.9 mmol/L), median (IQR) | 2.0 (1.0, 5.0) | 2.0 (1.0, 5.0) | 2.0 (1.0, 5.0) | 0.1 |
| In range (3.9-10.0 mmol/L), median (IQR) | 51.0 (37.0, 64.0) | 52.0 (38.0, 64.0) | 51.0 (37.0, 64.0) | 0.4 |
| Above range (>10.0 mmol/L), median (IQR) | 45.0 (31.0, 60.0) | 45.0 (31.0, 59.0) | 46.0 (31.0, 61.0) | 0.5 |
| **Glycated haemoglobin (HbA1c)**^b^ |  |  |  |  |
| Start HbA1c (mmol/mol), median (IQR) | 66.0 (56.0, 79.0) | 66.0 (56.5, 75.5) | 67.5 (56.0, 81.0) | 0.3 |
| Audit HbA1c (mmol/mol), median (IQR) | 61.0 (53.0, 72.0) | 60.0 (53.0, 72.0) | 61.5 (52.0, 70.0) | 0.9 |
| Change in HbA1c (mmol/mol), median (IQR) | -4.0 (-13.0, 3.0) | -3.0 (-11.0, 5.0) | -5.5 (-14.0, 2.8) | 0.1 |
|  |  |  |  |  |
| Notes: Not specified was the coding entry “Diabetes – not specified” within SystmOne and “Other” equals any other sub type of diabetes; ^a^ data available for 1218 patients (532 face-to-face and 686 online); ^b^ matched data available for 425 patients (207 face-to-face and 218 online); *p* indicates statisitical differences between onboarding methods and bold equals *p* < 0.05. | | | | |

| **Table S2:** Descriptive characteristics of the sample excluded and included within the analsyes. | | | |  |
| --- | --- | --- | --- | --- |
|  | **All**  **(n = 1261)** | **< 70% data**  **(n = 326)** | **≥ 70% data**  **(n = 935)** | ***p*** |
| **Age in years, median (IQR)** | 45.0 (32.0, 57.0) | 36.0 (27.0, 51.0) | 47.0 (34.0, 58.0) | **<0.001** |
| **Sex, n (%)** |  |  |  |  |
| Female | 615 (48.8%) | 169 (51.8%) | 446 (47.7%) | 0.2 |
| Male | 646 (51.2%) | 157 (48.2%) | 489 (52.3%) |  |
| **Diabetes type, n (%)** |  |  |  | 0.5 |
| T1DM | 1,043 (82.7%) | 271 (83.1%) | 772 (82.6%) |  |
| T2DM | 64 (5.1%) | 15 (4.6%) | 49 (5.2%) |  |
| Not specified & other | 154 (12.2%) | 40 (12.3%) | 114 (12.2%) |  |
| **Ethnicity, n (%)** |  |  |  | 0.1 |
| Asian | 161 (12.8%) | 47 (14.4%) | 114 (12.2%) |  |
| Black | 25 (2.0%) | 12 (3.7%) | 13 (1.4%) |  |
| Mixed | 316 (25.1%) | 79 (24.2%) | 237 (25.3%) |  |
| Other | 32 (2.5%) | 8 (2.5%) | 24 (2.6%) |  |
| White | 727 (57.7%) | 180 (55.2%) | 547 (58.5%) |  |
| **Deprivation score, n (%)** |  |  |  | **<0.001** |
| Deprivation group 1 (most) | 118 (9.4%) | 47 (14.4%) | 71 (7.6%) |  |
| Deprivation group 2 | 185 (14.7%) | 52 (16.0%) | 133 (14.2%) |  |
| Deprivation group 3 | 226 (17.9%) | 68 (20.9%) | 158 (16.9%) |  |
| Deprivation group 4 | 364 (28.9%) | 79 (24.2%) | 285 (30.5%) |  |
| Deprivation group 5 (least) | 368 (29.2%) | 80 (24.5%) | 288 (30.8%) |  |
| **Onboarding method, n (%)** |  |  |  | 0.8 |
| Face-toface | 560 (44.4%) | 147 (45.1%) | 413 (44.2%) |  |
| Online | 701 (55.6%) | 179 (54.9%) | 522 (55.8%) |  |
| **Libre metrics** |  |  |  |  |
| Libre duration in days, median (IQR) | 657.0 (394.0, 950.0) | 666.0 (399.2, 959.0) | 656.0 (394.0, 946.0) | 0.6 |
| Percent active, median (IQR) | 91.0 (68.0, 98.0) | 39.0 (17.0, 56.0) | 95.0 (89.0, 99.0) | **<0.001** |
| Scans per day, n (%) | 8.0 (5.0, 12.0) | 2.0 (1.0, 4.0) | 10.0 (7.0, 14.0) | **<0.001** |
| Average glucose (mmol/L) , median (IQR) | 10.0 (8.7, 11.7) | 11.3 (9.2, 14.4) | 9.8 (8.6, 11.1) | **<0.001** |
| Glucose Management Indicator (%), median (IQR)^a^ | 7.6 (7.1, 8.4) | 8.3 (7.4, 9.5) | 7.5 (7.0, 8.1) | **<0.001** |
| Coefficient of variation (%), median (IQR) | 38.5 (34.5, 43.0) | 40.3 (35.3, 46.0) | 37.9 (34.3, 42.2) | **<0.001** |
| Percent time in ranges |  |  |  |  |
| Below range (<3.9 mmol/L), median (IQR) | 2.0 (1.0, 5.0) | 3.0 (1.0, 6.0) | 2.0 (1.0, 5.0) | 0.3 |
| In range (3.9-10.0 mmol/L), median (IQR) | 51.0 (37.0, 64.0) | 39.0 (23.0, 55.0) | 54.0 (41.0, 66.0) | **<0.001** |
| Above range (>10.0 mmol/L), median (IQR) | 45.0 (31.0, 60.0) | 56.0 (39.0, 74.0) | 43.0 (30.0, 56.0) | **<0.001** |
| **Glycated haemoglobin (HbA1c)**^b^ |  |  |  |  |
| Start HbA1c (mmol/mol), median (IQR) | 66.0 (56.0, 79.0) | 71.0 (58.0, 88.0) | 66.0 (56.0, 76.0) | **0.02** |
| Audit HbA1c (mmol/mol), median (IQR) | 61.0 (53.0, 72.0) | 67.0 (56.0, 81.0) | 59.0 (52.0, 68.0) | **<0.001** |
| Change in HbA1c (mmol/mol), median (IQR) | -4.0 (-13.0, 3.0) | -2.0 (-11.0, 7.0) | -4.5 (-13.0, 2.0) | **0.03** |
|  |  |  |  |  |
| Notes: Not specified was the coding entry “Diabetes – not specified” within SystmOne and “Other” equals any other sub type of diabetes; ^a^ data available for 1218 patients (283 not included and 935 included); ^b^ matched data available for 425 patients (101 not included and 324 included); *p* indicates statisitical differences between groups and bold equals *p* < 0.05. | | | | |


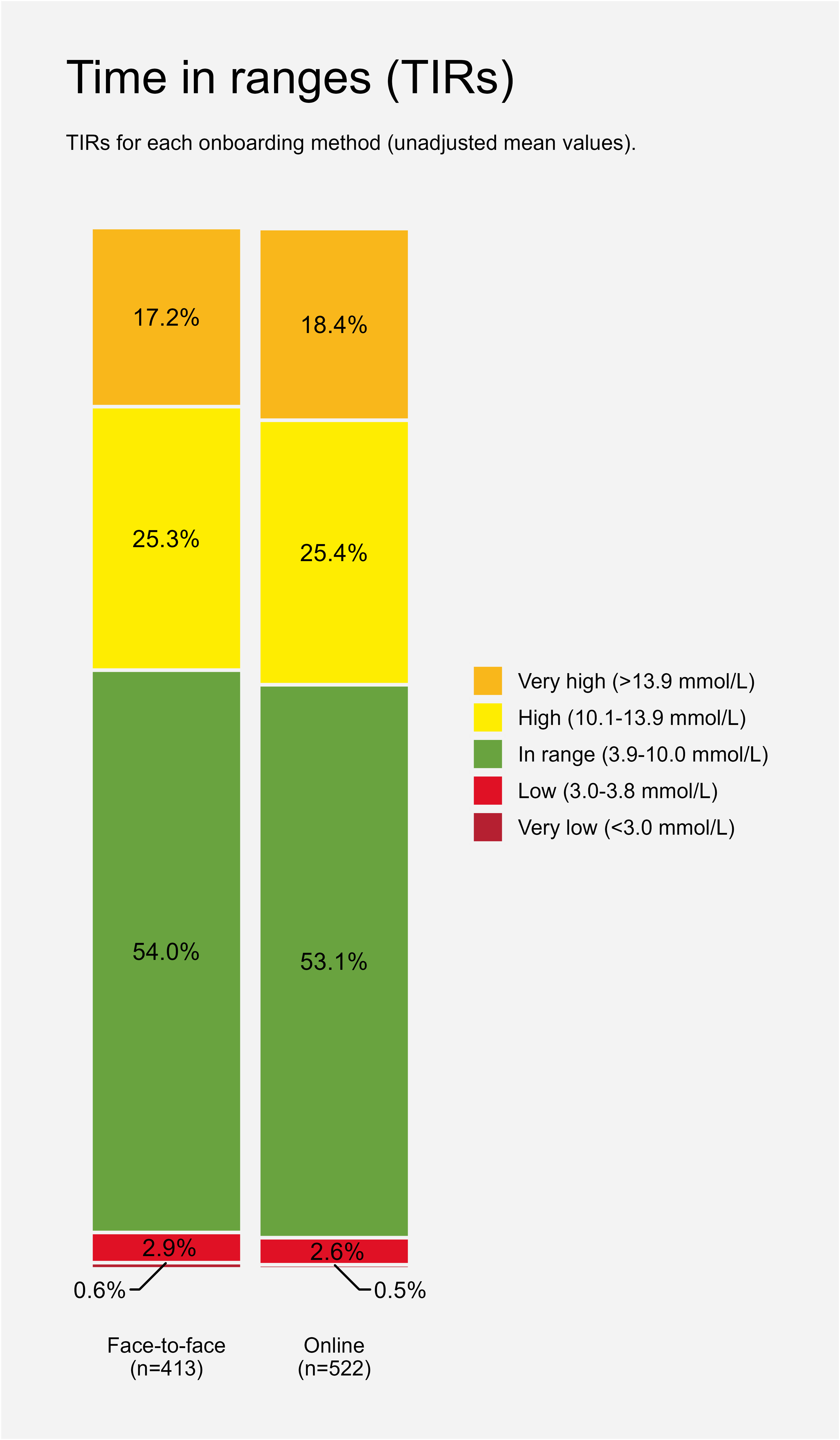


**Figure S1.** Unadjusted time in ranges (TIRs) for each onboarding group including level 2 categories.
